# Homeopathic Preparations to Control the Rosy Apple Aphid (*Dysaphis plantaginea* Pass.)

**DOI:** 10.1100/tsw.2010.12

**Published:** 2010-01-08

**Authors:** Eric Wyss, Lucius Tamm, Joachim Siebenwirth, Stephan Baumgartner

**Affiliations:** ^1^ Research Institute of Organic Agriculture FiBL, Frick, Switzerland; ^2^ Clinic for Homeopathy, Wolfratshausen, Germany; ^3^ Society for Cancer Research, Hiscia Institute, Arlesheim, Switzerland; ^4^ Institute of Complementary Medicine KIKOM, University of Berne, Switzerland

**Keywords:** aphid control, apple, homeopathy, Lycopodium clavatum, plant pest model system, nosode

## Abstract

A laboratory model system with the rosy apple aphid (*Dysaphis plantaginea* Pass.) on apple seedlings was developed to study the effects of homeopathic preparations on this apple pest. The assessment included the substance *Lycopodium clavatum* and a nosode of the rosy apple aphid. Each preparation was applied on the substrate surface as aqueous solution of granules (6c, 15c, or 30c). Controls were aqueous solutions of placebo granules or pure water. In eight independent, randomized, and blinded experiments under standardized conditions in growth chambers, the development of aphids on treated and untreated apple seedlings was observed over 17 days, each. Six experiments were determined to assess the effects of a strict therapeutic treatment; two experiments were designed to determine the effects of a combined preventative and therapeutic treatment. After application of the preparations, the number of juvenile offspring and the damage on apple seedlings were assessed after 7 and 17 days, respectively. In addition, after 17 days, the seedling weight was measured. In the final evaluation of the six strictly therapeutic trials after 17 days, the number of juvenile offspring was reduced after application of *L. clavatum* 15c (-17%, *p* = 0.002) and nosode 6c (-14%, *p* = 0.02) compared to the pure water control. No significant effects were observed for leaf damage or fresh weight for any application. In the two experiments with combined preventative and therapeutic treatment, no significant effects were observed in any measured parameter. Homeopathic remedies may be effective in plant-pest systems. The magnitude of observed effects seems to be larger than in models with healthy plants, which renders plant-pest systems promising candidates for homeopathic basic research. For successful application in agriculture, however, the effect is not yet sufficient. This calls for further optimization concerning homeopathic remedy selection, potency level, dosage, and application routes.

## INTRODUCTION

Pests limit yields and quality in agricultural plant production. One example of a yield- and quality-limiting pest is the rosy apple aphid (*Dysaphis plantaginea* Pass.), considered to be a major pest in apple orchards[1]. This aphid species causes irreversible damage to leaves, branches, and fruits.

In recent years, increasing levels of resistance to insecticides and concerns about pesticide residues in agricultural products have stimulated an increased interest of fruit growers in new control strategies. While considering new strategies, the question arises if homeopathic preparations could be of use to control this aphid or other pest species.

Generally, plants are able to react to potentized substances[2,3,4,5]. This capability of plants to react to homeopathy has mostly been proven by using healthy plants, but the observed differences to untreated plants were generally small. The reaction of diseased plants to homeopathic treatment should theoretically be more pronounced. However, only limited scientific data on the effects of homeopathic preparations on diseased plants are known[2,3,4,5,6,7,8,9] and some of these assessments lack statistical evidence. Therefore, scientifically sound model systems have to be developed in order to assess the potential of homeopathic treatments for plant pests and diseases. In a preliminary study, a plant-disease model with apple and apple scab (*Venturia inaequalis* Cke.) was developed for controlled laboratory trials[10]. It consisted of apple seedlings that were artificially inoculated with apple scab and treated with homeopathic preparations. This plant-disease model allowed assessments under standardized conditions and data complying with statistical requirements[11].

The objective of this study was to develop a similar plant-*pest* model system for the assessment of homeopathic preparations based on the experience with the plant-*disease* model. The apple seedlings were inoculated with one fundatrix of *D. plantaginea*. The offspring and the damage to leaves could easily be assessed. In repeated laboratory experiments, homeopathically prepared *Lycopodium clavatum* and a nosode of the rosy apple aphid were tested for possible impact on aphid offspring and leaf damage.

## MATERIALS AND METHODS

### Selection of Homeopathic Preparations

Based on previous experiments[10], two types of preparations were included in the assessments with the new plant-pest model system: the specially selected homeopathic remedy *L. clavatum* and a nosode of the rosy apple aphid.

As there is no systematically compiled *Materia medica* for plants, it is not possible to approach diseased plants by the classical homeopathic remedy selection procedure. Especially, a thorough anamnesis, a crucial tool to extract the characteristics of a case, is not possible. Therefore, we tried to extrapolate from the observed plant disease patterns to similar human symptoms, and to select corresponding remedies with the aid of the Complete Millenium Repertory (MacRepertory Pro V. 7.5.6.1, Kent Homeopathic Associates Inc., San Rafael, CA). The repertorization ended up with four potential remedies for the treatment of the rosy apple aphid: *L. clavatum*, *sulfur*, *Mercurius solubilis*, and *Natrium muriaticum*. All are used for constitutional therapies, but are also important acute remedies for a great number of diseases. For feasibility reasons, only one remedy – *L. clavatum*, being the most evident – was chosen for the present study.

To complement this classical homeopathic remedy, a nosode of the rosy apple aphid was chosen as another therapeutic approach for the apple seedlings.

Both preparations were applied in three potency levels only (6c, 15c, and 30c) due to restrictions in laboratory, manpower, and financial resources. These potency levels were chosen based on experiences in human therapy and plant experimentation[4,5].

## Homeopathic Preparations and Controls

All homeopathic preparations were prepared as centesimal potencies (with a 1:100 dilution ratio, supplied as granules) by the company Spagyros AG (Gümligen, Switzerland). The homeopathic preparations were first potentized in the solid phase (triturated) for three or six potency levels, then potentized in the liquid phase, and finally impregnated on sugar granules. Solid potentization (trituration) was performed by hand using a porcelain mortar and pestle (Haldenwanger, Berlin, Germany). To obtain a potency level z, 62 mg of potency level z–1 were triturated in 2 g lactose (Hänseler, Herisau, Switzerland) for 20 min (6 min grinding, 4 min scraping, 6 min grinding, 4 min scraping). To this mixture, 2 g lactose were added and triturated analogously for 20 min. This procedure was repeated with another 2 g lactose to finally obtain 6 g of potency level z. Liquid potentization was done by hand in brown glass bottles of hydrolytical class 3 (Müller & Krempel, Bülach, Switzerland). To obtain a potency level z, 1 part of potency level z–1 was added to 99 parts of dilution medium and successed by 60 firm vertical beats against a leather cushion. Liquid potentization medium was either water (aqua purificata, TKA-Pacific, TKA Wasseraufbereitungssysteme GmbH, Niederelbert, Germany) or mixtures of water with ethanol 94% (Eidgenössische Alkoholverwaltung, Bern, Switzerland). Water was used for the first potency step after trituration, 30% ethanol for the second potency step after trituration, 43% ethanol for all other potency levels, except for the last potency level (for granule impregnation) where 73% ethanol was used.

For the nosode preparations, leaves of apple seedlings with typical symptoms and aphids were triturated in lactose up to 3c, and further potentized in liquid potentization medium as described above. *L. clavatum* (Dixa, St. Gallen, Switzerland) was triturated up to 3c or 6c (for the 6 or 15/30c granules, respectively), and further potentized in liquid potentization medium as described above.

Granules were prepared in the potency levels 6c, 15c, and 30c; 100 g of granules size 2 (made of glucose and invert sugar; Werner, Tornesch, Germany) were impregnated with 1 ml of the corresponding liquid potency level in a rotating drum for 15 min. Placebo granules were of identical origin, but not impregnated. Granules were stored in separate brown glass vials.

A person not involved in the experiments (S.B.) coded all homeopathic preparations, including placebo granules, by assigning a random letter code to the brown glass vials used for storage of the granules. Codes were revealed only after all experiments and statistical analyses were accomplished.

## Choice of Controls

Treatment was applied by dissolving 10 homeopathic sugar granules in 250 ml tap water and watering the plants with these solutions. We used two sorts of controls. The first control consisted of remedy-untreated seedlings (treatment with tap water only), since this type of control is the official standard in plant-pest experimentation according to the European and Mediterranean Plant Protection Organization (EPPO) standards[11]. As a second control (carrier control), we used tap water with dissolved untreated granules (not impregnated with successed or potentized ethanol or lactose, respectively). This type of control was chosen to account for any effects of the carrier substance (sugar). Specificity of any homeopathic treatment effects could be controlled by comparing the effects of both potentized substances at the potency levels used.

## Aphids and Apple Seedlings

In Spring 2008, fundatrices of *D. plantaginea* were sampled in organic apple orchards in northwestern Switzerland. They were transferred to apple seedlings and reared in climatic chambers at constant conditions (temperature 12ºC; photoperiod 13.5L:10.5D; photosynthetic active radiation 250 µmol photons/$m^2$sec). For each experiment new offspring of the same age were used.

Apple seedlings of the variety Golden Delicious were planted in pots (diameter 0.1 m) with standard substrate (Einheitserde Typ 0; Einheitserde, Germany). For each experiment, new seedlings at the stage of five to six leaves were used.

## Plant-Pest Model System

The model system consisted of a potted apple seedling that was transferred in a transparent plastic tube (diameter 0.1 m) and put on a saucer to prevent contact of differently treated seedlings. On each apple seedling, a young fundatrix was inoculated (with the exception of a mock-inoculated quality check). For the treatments, 10 granules of each homeopathic preparation (or untreated placebo granules) were diluted in 250 ml tap water and pivoted until granules were dissolved. Twenty (20) ml of these solutions were pipetted (20 ml single-serving syringe for each treatment) on the substrate surface of the potted seedlings. Control seedlings got 20 ml tap water.

All experiments were conducted in climatic chambers (GroBank, CLF PlantClimatics GmbH) with standardized conditions (temperature 12ºC; photoperiod 13.5L:10.5D; photosynthetic active radiation 250 $\mu$mol photons/$m^2$sec).

## Experiments with Therapeutic Treatments

The objective of the six identical, blinded experiments was to assess the effects of therapeutic treatments with *L. clavatum* (6c, 15c, 30c, or placebo granules) and the nosode (6c, 15c, 30c, or placebo granules) on the offspring of aphids, on leaf damage, and on seedling weight. Water-treated seedlings served as main control. Three days after the transfer of the fundatrices, the seedlings were treated for the first time; three additional treatments followed in a 3- to 4-day interval.

Each experiment consisted of a completely randomized block design with 10 replicates (seedlings). Thus, each experiment included 100 plants in total (10 seedlings × 10 treatments: three potency levels of *L. clavatum*, three potency levels of the nosode, two placebo granules treatments, one control treated with water only, and one uninfected [mock-inoculated] negative control).

The six experiments were repeated in a 14-day interval between April 21 and June 19 in 2008. To show any treatment effects, the number of juvenile offspring and the damage on apple leaves (percentage of total leaf surface) were assessed after 7 and 17 days. In addition, the fresh weight of apple seedlings (without roots) was measured after 17 days.

## Experiments with Combined Preventative and Therapeutic Treatment

In contrast to the first experimental setup, the objective of the two identical, blinded, and repeated experiments was to assess the effects of a combined preventative and therapeutic treatment with *L. clavatum* (6c, 15c, 30c, placebo) and the nosode (6c, 15c, 30c, placebo) on the offspring of aphids, on leaf damage, and on seedling weight. Water-treated seedlings served as control. Three days before the transfer of the fundatrices, the seedlings got one preventative treatment; three therapeutic treatments followed in a 3- to 4-day interval.

The experimental design and assessments were identical as in the first experimental setup. The two experiments were carried out in a 14-day interval between May 13 and June 16 in 2008.

## Statistical Analysis

Data of the final assessments (after 17 days) were evaluated for statistical significance based on two-way analysis of variance (ANOVA) F tests with $\alpha$ = 5%. Independent ANOVA factors were (1) treatment (n =

9: one control, two placebos, and six homeopathic preparations; the mock-inoculated quality check was excluded before data analysis), and (2) experimental number (n = 6 or 2). Dependent variables were the number of juvenile offspring, leaf damage, and fresh weight. Data on juvenile offspring were square root transformed prior to statistical analysis to achieve a normal data distribution. The LSD test was used for planned comparison of treatment means with the untreated water control ($p = 0.05$); according to EPPO Standards (2004), the untreated control has to be the main reference[11]. Planned comparisons were evaluated with the LSD test only if the preceding F test was significant ($p < 0.05$). This procedure (protected Fisher's LSD) gives a good safeguard against type I error without being too conservative, i.e., it also gives good security against type II error[12].

Leaf damage data of the preliminary assessments after 7 days were square root transformed prior to statistical analysis to achieve a normal data distribution. Data were evaluated for statistical significance based on two-way ANOVA F tests with $\alpha = 5\%$ analogously as for the final assessment after 17 days as outlined above. The number of juvenile offspring after 7 days was evaluated with the Chi-square test.

All analyses were made using the software STATISTICA Version 6 (Stat Soft, Inc., Tulsa, OK).

## RESULTS

### Plant-Pest Model

The eight experiments with therapeutic and combined preventative/therapeutic treatments of apple seedlings to control the offspring of rosy apple aphids could be realized under standardized conditions. Less than 1% of the seedlings had to be excluded from data analysis, in all cases due to wrong manipulations during treatments. However, a technical problem might have enhanced the variability of results. The age of apple seedlings and fundatrices used in the experiments varied between the experiments: ±4 days for apple seedlings, ±2 days for the rosy apple aphids. Otherwise, no additional technical problem occurred with this new plant-pest model system. None of the mock-inoculated plants bore any rosy apple aphids. Thus, there was no cross-contamination between the plants regarding aphids within each experiment.

### Preliminary Assessment: Effects of Therapeutic Treatments after 7 Days

Data of all six experiments were analyzed jointly. In 34.4% of all cases, no juvenile offspring were observed. Thus, the number of juvenile offspring was strongly non-Gaussian; we therefore did not apply parametric analysis of variance to the data. The proportion of seedlings without and with juvenile offspring did not differ between the treatments ($p = 0.78$, Chi-square test). No significant differences were observed between treated and untreated apple seedlings for the percentage of damaged leaves (two-way ANOVA, $p = 0.57$ for the factor treatment, F test, square root transformed data). Furthermore, we did not observe any fundatrix that left an apple seedling.

### Final Assessment: Effects of Therapeutic Treatments after 17 Days

To investigate the efficacy of therapeutic treatments with homeopathic preparations, data of the six experiments were analyzed jointly in a two-way ANOVA with the independent factors "treatment" (nine parameters) and "experiment number" (six experiments). The dependent variables (percentage of damaged leaves, juvenile number, and fresh weight) were analyzed separately. In all three cases, the factor "experiment number" yielded highly significant effects ($p < 0.001$). This reflects the differences in absolute values between single experiments, due to differences in age of apple seedlings and fundatrices between the experiments as discussed above. The results regarding the factor "treatment" (six homeopathic preparations and three controls) are discussed in the following paragraphs.

No significant differences were observed between treated and untreated apple seedlings for the percentage of damaged leaves (Fig. 1A, $p = 0.84$, F test for factor "treatment").

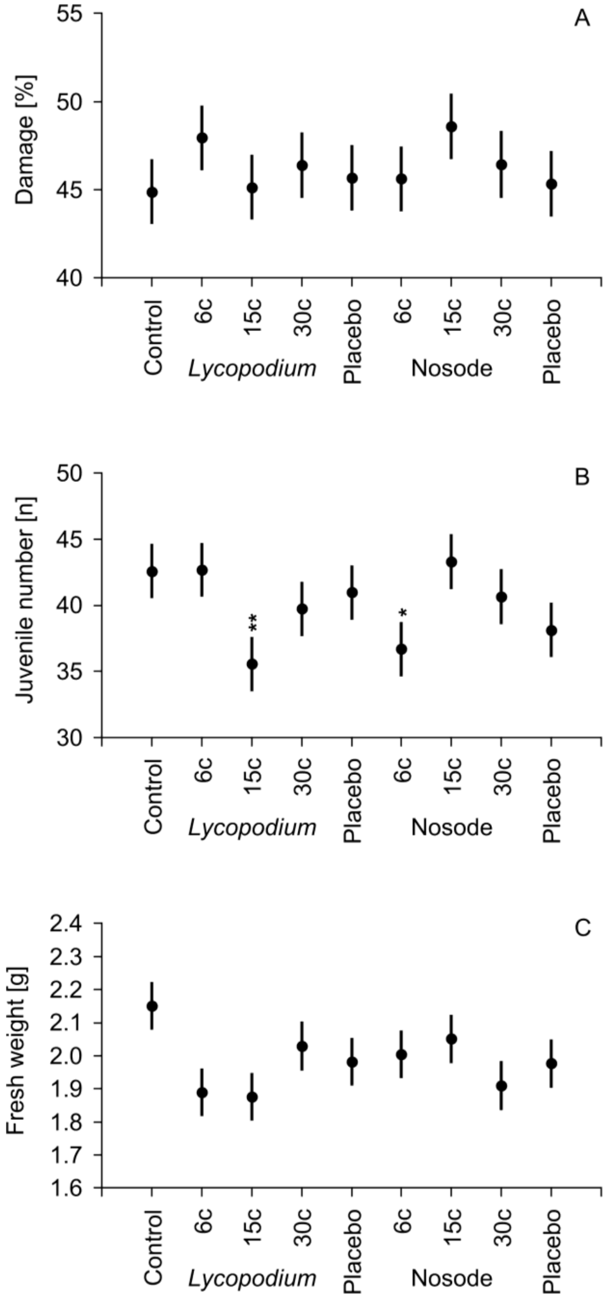

**FIGURE 1**. Effects of a curative homeopathic treatment on apple seedlings inoculated with the rosy apple aphid: final assessment of *L. clavatum* or a nosode at the potency levels 6c, 15c, or 30c, or placebo granules after 17 days. (A) Percentage of damaged leaves; (B) number of juvenile offspring (n); (C) fresh weight (g). Mean values ± standard errors of six independent, randomized, and blinded experiments. Statistical evaluation with LSD test, compared to the untreated control: $^{**}p < 0.01$, $^{*}p < 0.05$.

However, there were some treatment effects concerning the number of juvenile offspring (Fig. 1B, $p = 0.005$, F test for factor “treatment”, square root transformed data). The two placebo controls were not significantly different from the untreated water control. Therefore, the effect of the granule carrier substance (glucose and invert sugar) seems to be negligible. However, the two treatments – *L. clavatum* 15c ($p = 0.002$, LSD test of square root transformed data) and nosode 6c ($p = 0.02$, LSD test, square root transformed data) – significantly differed from the untreated water control (Fig. 1B). Compared to the water control, juvenile offspring were reduced by 17% under *L. clavatum* 15c treatment and by 14% under nosode 6c. Numerically, *L. clavatum* 15c reduced the number of juvenile offspring in all six experiments, while the nosode 6c reduced them in five of six experiments. The absolute number of juvenile offspring varied between experiments. This effect allowed us to observe that the treatment effects of *L. clavatum* 15c and nosode 6c were stronger (up to 30% offspring reduction) with less absolute number of juvenile offspring on the apple seedlings (Fig. 2).

No significant differences in fresh weight were found between treated and untreated apple seedlings (Fig. 1C, $p = 0.062$, F test for factor “treatment”). However, there was a tendency for reduced fresh weight for all treated apple seedlings.

### Effects of Combined Preventative and Therapeutic Treatments

Data of the two experiments were analyzed jointly in two-way ANOVA. No significant differences were observed between treated and untreated apple seedlings for the percentage of damaged leaves, number of juvenile offspring, and fresh weight of apple seedlings for any assessment time.

## DISCUSSION

Based on the hypothesis that diseased organisms should show a more evident reaction to treatments with homeopathic preparations than healthy organisms, the first objective of this study was to develop an easy-to-handle plant-pest model. It should serve to screen homeopathic preparations under constant climatic conditions in the laboratory. Data gained with this model system should serve as the basis for further experiments under field and farm conditions. Several considerations led to the tested model system: (1) even if leaf disks are easy to handle and allow a greater number of replicates[13], a total plant model would be closer to natural conditions; (2) effects of homeopathic preparations on diseased plants might be more easily detected in perennial plants due to their longer life; (3) pests should be used as model organisms that cause important, but not lethal, damage; (4) the model system should allow statistically reproducible datasets. Apples are a perennial crop with several important pests that cause severe damage. Apple seedlings are juvenile, total plants that can easily be produced and are used as a model for pesticide screening[14]. On apple seedlings, the rosy apple aphid rapidly causes visible, drastic damage on leaves. Therefore, the rosy apple aphid on apple seedlings was chosen as a potential plant-pest model to test homeopathic therapies.

We were very satisfied with the handling of this plant-pest model and could not identify any major disadvantage associated with this experimental setup. In addition, we observed no cross-contamination between plants regarding aphids, which reduced the probability of erroneous results. The mean coefficient of variation was 34% for leaf damage, 25% for the number of juvenile offspring (square root transformed), and 31% for fresh weight at the final assessment after 17 days. In future experiments, this rather high variability might be reduced by an even more homogenous plant and pest age selection. Nevertheless, a model system with interacting organisms will always show a certain range of variability. Due to this fact, the statistical power of this type of model system will fail to show small differences in efficacy (less than 10%) of tested homeopathic remedies.

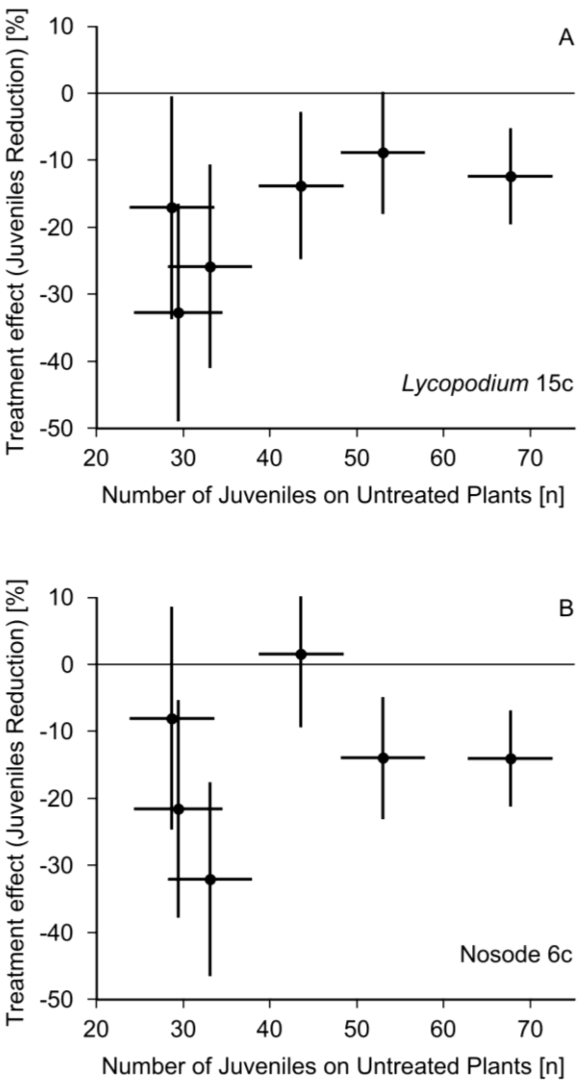

**FIGURE 2**. Treatment effect size as a function of the absolute pest number for the six individual curative experiments. Treatment effect size of *L. clavatum* 15c (A) or nosode 6c (B) is plotted as percent juvenile offspring reduction on the y-axis (mean ± SE on y-axis) as a function of the number of juveniles on untreated control plants on the x-axis (mean ± SE on x-axis). Absolute pest number varied from experiment to experiment due to differences in the age of fundatrices. SE are based on the measurement values of the 10 replicates (apple seedlings) within each experiment.

The second objective of this study was to assess the efficacy of two specifically selected homeopathic preparations to control the rosy apple aphid. By repeated therapeutic treatments with *L. clavatum* 15c and the nosode 6c, the juvenile offspring could significantly be reduced compared to the pure water control. The reduction of juvenile offspring by 17 and 14%, respectively, is not relevant for agricultural practice, however. But, compared to studies with healthy plants[15,16,17] that document effects of homeopathic preparations in the order of 1–3% on growth[4,18], the resulting treatment effect of the homeopathic preparations was more pronounced in our pest-plant system. Thus, the latter might be an interesting tool for homeopathic basic research. This general result is in line with other toxicological and phytopathological investigations, which all showed homeopathic treatment effects in the order of 10–20%[13,19,20,21].

Due to lack of resources, we were not able to implement systematic negative control experiments[22] to document system stability continuously and to ensure appropriateness of statistical evaluation empirically. Although significance levels were quite low and although effects of *L. clavatum* 15c were numerically reproducible over all six single trials with curative treatment, one might argue that the effects found were artifacts (false-positive results). For future use in homeopathic basic research, systematic negative control experiments are therefore highly recommended to control system variability.

The homeopathic remedies were prepared according to standard procedures: after trituration in the solid phase, further potency levels were prepared by manual succussion in water/ethanol mixtures. Finally, granules of the potency levels 6c, 15c, and 30c were prepared by impregnation with the corresponding liquid potency level. Granules were stored light-protected in standard brown glass vials until use. For plant application, 10 granules of each homeopathic preparation (or untreated placebo granules) were diluted in 250 ml tap water and pivoted until granules were dissolved. 20 ml of these solutions were pipetted on the substrate surface of the potted seedlings. We have no idea whether this application route is optimal for the treatment of plants. Other ways to prepare the homeopathic potencies (e.g., direct potentization in water without using ethanol or granules) or to apply them to the plants (e.g., by foliar spray instead of root application, by another dosage [amount and frequency]) should be evaluated in future experiments. Furthermore, other remedies should be tested. In addition to *L. clavatum*, *sulfur*, *M. solubilis*, and *N. muriaticum* were also promising candidates, but could not be tested due to lack of resources. Analogously, apart from 6c, 15c, and 30c, other potency levels could be screened for larger efficiency.

Based on our main interest to determine the potential of homeopathic preparations for plant protection, we used two sorts of control groups. The first control consisted of remedy-untreated seedlings (treatment with tap water only), since this type of control is the official standard in plant-pest experimentation according to the EPPO standards[11]. As a second control (carrier control), we used tap water with dissolved untreated granules (not impregnated with succussed or potentized ethanol or lactose, respectively). This type of control was chosen to account for any effects of the carrier substance (sugar) present in traces in the watering solution. From the point of view of homeopathic basic research, the "perfect" control would be triturated lactose, potentized analogously to the verum preparations and impregnated on granules. Our main interest in this experiment was to determine the potential of homeopathic preparations for plant protection, however, and therefore we chose the controls mentioned above. When conducting further experiments with this system for basic research questions, we propose to use potentized lactose as the most appropriate control.

After these six experiments on the therapeutic effect of the two preparations on the control of the rosy apple aphid, the mode of action is not clear. Only the repellent effect on aphids can be excluded, as none left the treated apple seedlings. If the lower number of offspring is due to effects on fertility or on slowing down the development of aphids, it is not yet known.

All experiments have been done under identical, standardized climatic conditions. However, the timing of the rearing of apple seedlings and rosy apple aphids brought some difficulties: the age of the apple seedlings and the fundatrices used varied between (not within) the experiments. What first looks like a drawback can be easily dealt with through application of two-way ANOVA. It furthermore revealed the important information that the effect of the homeopathic preparations was stronger (up to 30% juvenile inhibition) for experiments with a lower absolute number of juvenile offspring ($n \approx 30$ on the control plants), compared to about 10% offspring inhibition for experiments with a larger juvenile offspring ($n > 40$ on the control plants, cf. Fig. 2). This means that the capacity of the system to react to homeopathic preparations was at its upper limit. Consequently, the model system might be improved by using bigger, 1-year-old apple plants that might be hypothesized to have a stronger self-regulation to cope with the seemingly rather severe aphid attack.

Contrary to the number of juvenile offspring, no differences between treatments were found for leaf damage. Usually, the number of rosy apple aphids and the damage are positively correlated[23]. A possible reason for the contradiction in these experiments might be that the homeopathic preparations have no immediate effect on the rosy apple aphid. Therefore, the damage of the one fundatrix and her offspring might have surpassed the damage threshold for these apple seedlings within the 17-day test period. This model system is perfectly adapted to show effects of immediately active agents against aphids[24], but it might fail to show differences when treatments have a retarded effect, e.g., induced by the stimulation of self-defense mechanisms of plants. As mentioned above, the model system should be adapted by using older apple plants that would be able to recover from a severe aphid attack. It then might be possible to observe also a retarded effect of treatments with homeopathic preparations on leaf damage.

No statistically significant effect of combined preventative and therapeutic treatments could be observed within the two serial experiments. This may be due to the fact that the statistical power of these two experiments is lower than the power of the six experiments with therapeutic treatment only. However, the trends seen in juvenile number in the experimental groups of *L. clavatum* 15c (2% decrease compared to the water control) and of the nosode 6c (7% increase) are not in line with the results of the strictly therapeutic trials. Despite the lower statistical power of the two experiments, one can at least raise the hypothesis that an additional prophylactic treatment does not seem to increase the response of the system considerably; the additional prophylactic treatment rather seems to counteract the curative treatments. It seems premature to us to speculate about a possible correspondence of our results to the widespread view that the main strength of homeopathy is rather disease treatment than specific disease prophylaxis.

## CONCLUSIONS

According to our results, homeopathic remedies may be effective in plant-pest systems. The magnitude of observed effects is larger than in models with healthy plants, which renders plant-pest systems attractive for homeopathic basic research. The larger effect size has to be put into relation with the enhanced variability and the larger expenditures for handling and cultivation, however. In addition, the effect size is not yet sufficient for successful application in agriculture. This all calls for further optimization of this model system regarding homeopathic remedy selection, potency level, dosage (amount and frequency), application form (e.g., granules or dilution), and application routes (e.g., foliar spray vs. root treatment). Finally, the use of systematic negative control experiments is to be recommended in future investigations to control system variability.

## ACKNOWLEDGMENTS

The authors thank S. Stutz, S. Bütler, and D. Flückiger for laboratory assistance and the Karl and Veronica Carstens Foundation for financing the project. We thank C. Scherr and J. Clausen for critical comments on an earlier version of the manuscript. The production of the homeopathic preparations by the company Spagyros AG is gratefully acknowledged. Neither the authors nor their affiliates have any conflicts of interest to declare.
